# Unlocking Methane Generation via Photo‐Thermal‐Coupled CO_2_ Hydrogenation by Integrating FeNiCrMnCo Multicomponent Alloy with GaN Nanowires

**DOI:** 10.1002/advs.202501298

**Published:** 2025-04-17

**Authors:** Muhammad Salman Nasir, Ying Zhao, Haotian Ye, Jinglin Li, Ping Wang, Ding Wang, Xinqiang Wang, Jun Song, Zhen Huang, Baowen Zhou

**Affiliations:** ^1^ Key Laboratory for Power Machinery and Engineering of Ministry of Education Research Center for Renewable Synthetic Fuel School of Mechanical Engineering Shanghai Jiao Tong University 800 Dongchuan Road Shanghai 200240 China; ^2^ Department of Mining and Materials Engineering McGill University 3610 University Street Montreal QC H3A0C9 Canada; ^3^ State Key Laboratory of Artificial Microstructure and Mesoscopic Physics School of Physics Nano‐Optoelectronics Frontier Center of Ministry of Education (NFC‐MOE) Peking University Beijing 10087 China; ^4^ Peking University Yangtze Delta Institute of Optoelectronics Nantong Jiangsu 226010 China; ^5^ Collaborative Innovation Center of Quantum Matter School of Physics Peking University Beijing 100871 China

**Keywords:** CO_2_ methanation, GaN, multicomponent alloys, photo‐thermal‐coupled

## Abstract

The exploration of a noble‐metal‐free photo‐thermal‐coupled catalytic architecture plays a vital role in solar‐driven conversion of carbon dioxide (CO_2_) into high‐value fuels and chemicals. In this study, FeNiCrMnCo multicomponent alloy (MCA) is integrated with GaN nanowires (NW's) for photo‐thermal‐coupled catalytic CO_2_ methanation. The MCA/GaN NW's nanohybrid demonstrates a considerable methane production rate of 199 mmol∙g^−1^∙h^−1^ with an impressive selectivity of 93% under white light irradiation of 3 W∙cm^−2^ at 290 °C by external heating. The turnover number approaches 20,160 mole CH_4_ per mole of MCA over a continuous operation period of 120 h, showcasing remarkable stability. Mechanistic investigations reveal that the unique MCA provides a flexible platform for tailoring the electronic and catalytic properties to optimize the adsorption and activation of CO₂ and H₂, thus leading to efficient and selective CO₂ methanation. This study presents an industry‐friendly architecture for photo‐thermal‐coupled CO_2_ hydrogenation into high‐value fuels and chemicals by coupling a noble‐metal‐free multicomponent alloy with GaN NWs, paving the way for advancements in sustainable energy conversion through CO_2_ utilization.

## Graphical abstract

A noble‐metal‐free FeNiCrMnCo multicomponent alloy (MCA) integrated with GaN nanowires achieves efficient photo‐thermal‐coupled CO₂ methanation, delivering a methane production rate of 199 mmol∙g^−^¹∙h^−^¹ with 93% selectivity. The system demonstrates exceptional stability and tunable catalytic properties, offering a sustainable pathway for solar‐driven CO₂ conversion into high‐value fuels and chemicals.

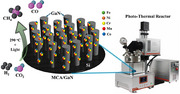

## 1 Introduction

The escalating concentration of CO_2_ in the atmosphere poses a critical threat to sustainable development.^[^ ^1^ ^]^ The conversion of CO_2_ using green hydrogen into green fuels and valuable chemicals showcases an ultimate solution for addressing this grand challenge.^[^ ^2^ ^]^ Among a vast variety of products, CH_4_ has increasingly attracted research interest owing to its high energy density and broadly available infrastructures for storage, transportation, and distribution.^[^ ^3^ ^]^ However, it is extremely challenging to achieve high activity and selectivity of methane generation from CO_2_ hydrogenation because of the chemical inertness of carbon dioxide and complex reaction networks.^[^ ^4^ ^]^ Traditionally, thermal catalytic methods often require high temperatures and elevated pressures, which necessitate extensive energy inputs and suffer from undesired side reactions, coke deposition, as well as metal sintering under harsh reaction conditions.^[^ ^5^ ^]^ What is more, rare and expensive metals are often required to achieve practically appreciable performance.^[^ ^6^ ^]^ There is an urgent demand to explore innovative approaches to advance the viability of CO_2_ methanation using earth‐abundant materials.^[^ ^7^ ^]^

Compared to the conventional thermo‐catalytic process, photo‐thermal‐coupled catalysis holds promise for overcoming the thermodynamic and kinetic limitations of CO_2_ methanation by coupling light irradiation with external heating. Solar‐driven hydrogenation of carbon dioxide offers a sustainable pathway for energy conversion and environmental remediation.^[^ ^8^ ^]^ By utilizing renewable solar energy, it provides a clean alternative to fossil fuel‐based processes, reducing dependence on nonrenewable resources. The integration of photo‐thermal catalysis enhances energy efficiency by combining light and heat, improving reaction selectivity and performance. This technology addresses environmental challenges by converting CO_2_, a major greenhouse gas, into valuable fuels like methane, contributing to carbon neutrality and climate change mitigation. Its versatility enables the production of various hydrocarbons, making it a flexible solution for energy storage and chemical synthesis. With its scalability and alignment with global clean energy goals, solar‐driven CO_2_ hydrogenation emerges as a transformative strategy for a sustainable future.^[^ ^7^, ^9^ ^]^ More specifically, photo‐thermal techniques enable the synergy between charge carriers and heat for simultaneously activating both CO_2_ and H_2_. The exploration of a rational multifunctional catalytic architecture by assembling appropriate active sites with a photo‐thermal platform is at the core.

MCAs, composed of five or more principal elements not necessarily in Equi atomic ratios, exhibit a unique combination of structural complexity. This distinctive multimetallic composition allows MCAs to tailor the electronic environment at the atomic scale without rare metals.^[^ ^10^ ^]^ Moreover, by leveraging the synergistic interactions between various metal constituents, MCAs enables flexible manipulation of the adsorption and dissociation of CO₂ and H₂ molecules, thus providing a flexible window for mediating CO_2_ hydrogenation toward CH_4_.^[^ ^11^ ^]^ Additionally, their exceptional thermal and chemical stability under harsh conditions further position MCAs as ideal candidates for activating and transforming CO₂ into high‐value products.^[^ ^12^ ^]^

Metal (hydro) oxides are the most extensively studied supports for loading cocatalysts to catalyse CO_2_ methanation.^[^ ^13^ ^]^ Nevertheless, they are often limited by the undesirable formation of bicarbonate, resulting in unsatisfactory activity, selectivity, and stability.^[^ ^14^ ^]^ By contrast, metal nitrides present an emerging platform for enhancing CO_2_ adsorption and activation because of the unique surface properties that avoids the formation of bicarbonate,^[^ ^15^ ^]^ thus promoting CO_2_ catalysis. Of note, in our recent studies,^[^ ^16^ ^]^ 1D gallium nitride (GaN) nanowires (NWs) supported by wafer‐scale silicon present an ideal building block for assembling a photo‐thermal‐coupled catalyst owing to the distinctive optoelectronic, thermal, and catalytic properties. More specifically, this binary semiconductor hybrid effectively harvests a broad spectrum of sunlight, utilizing high‐energy UV photons to generate charge carriers while simultaneously harnessing the photo‐thermal effect of visible and infrared light to locally heat the photocatalyst. This dual functionality not only enhances the efficiency of solar energy conversion but also improves the thermodynamic driving force for CO_2_ hydrogenation.

Inspired by these considerations, we present a noble‐metal‐free catalytic architecture for photo‐thermal‐coupled CO_2_ methanation by embedding FeNiCrMnCo MCA onto GaN nanowire arrays supported by wafer‐scale silicon (denoted as MCA/GaN NWs). The multiple composition of the FeNiCrMnCo MCA provides a flexible window to optimize the electronic interactions with CO₂ and H_2_. In combination with the unique properties of GaN NWs, this as‐prepared multifunctional architecture has thus yielded an impressive methane production rate of 199 mmol∙g^−1^∙h^−1^ with a remarkable selectivity of 93% by a combined effect of concentrated light intensity (3 W.cm^−2^) and external heating (290 °C), far outperforming the performance of pure thermo‐catalysis (Table S1, Supporting Information). Notably, a distinct TON of 20 160 mole CH_4_ per mole of MCA is achieved over 120 h, demonstrating the stability of the MCA/GaN wafer under continuous operation. Control experiments, in situ spectroscopic characterizations, and density functional theory calculations reveal that the MCA/GaN system promotes the formation of CH₄ through the stabilization of the key HCOO* intermediate with a lowered energy barrier. This study underscores the promise of integrating a noble‐metal‐free MCA with a GaN semiconductor platform for efficient and selective CO_2_ hydrogenation into green fuels and value‐added chemicals, marking a significant advancement in the field of sustainable energy production.

## 2 Results and Discussion

### 2.1 Morphological and Structural Characterization

**Figure 1:**
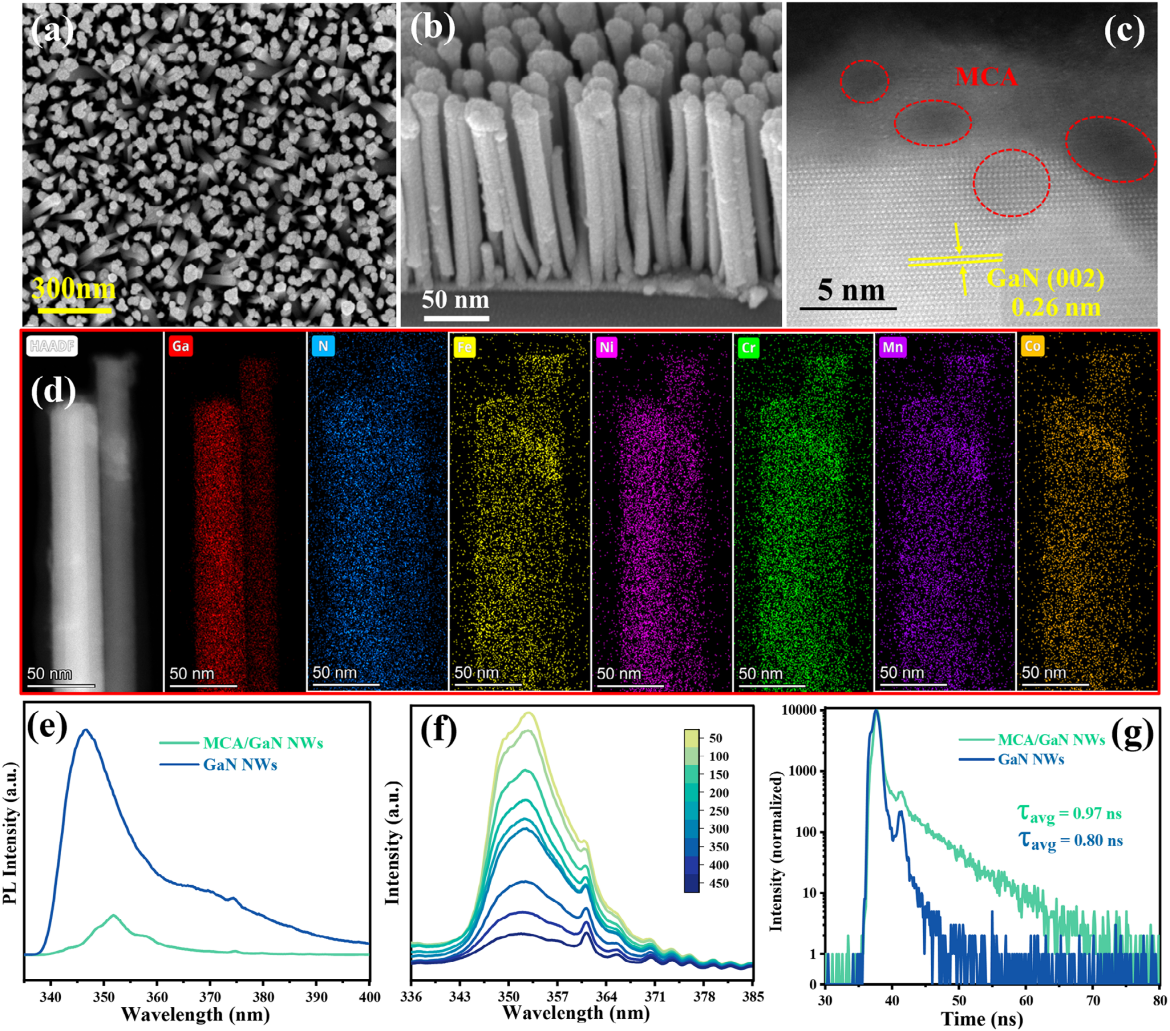
SEM images; a) Top View of GaN NWs; b) 25o tilted image of MCA‐decorated GaN NWs supported by silicon wafer; c) HRTEM image of MCA‐decorated GaN NWs; d) HAADF‐STEM and elemental mapping images of MCA‐decorated GaN NWs; e) Photoluminescence (PL) spectroscopy of MCA/GaN NWs; f) Temperature‐dependent photoluminescence spectroscopy of MCA/GaN NWs; g) Time‐resolved PL (TRPL) of GaN NWs and MCA/GaN NWs.

The plasma‐assisted molecular beam epitaxy (MBE) technique was used to controllably produce 1D GaN NWs on a 4‐inch silicon wafer (**Figure** **1**a) under N‐rich circumstances as reported in our previous studies.^[^ ^16^, ^17^ ^]^ The scanning electron microscopy (SEM) characterization reveals that GaN NWs on silicon wafers are arranged in a vertical alignment with a vertical height 500–600 nm (Figure 1b; Figure S1, Supporting Information). The lateral diameters range from 50 to 60 nm (Figure S2, Supporting Information). The 1D structure gives GaN NWs a large specific surface area, which is very helpful for increasing photon absorption, reducing charge carrier diffusion distance, and maximizing the exposure of catalytic sites.^[^ ^17^ ^]^ The simple photodeposition method was adopted to load five metals over the GaN NWs scaffold. The 1D uniform structure remains unchanged even after the introduction of MCA, as seen in Figure 1b. The energy dispersive X‐ray spectroscopy (EDX) results confirm the presence of Fe, Ni, Cr, Mn, and Co over the GaN NWs (Figure S4, Supporting Information). The TEM image (Figure S3a, Supporting Information) shows that the surface of GaN NWs was found to be uniformly distributed with MCA NPs (Figure S3a, Supporting Information). The high‐resolution transmission electron microscope (HR‐TEM) image in Figure 1c depicts a lattice spacing of 0.26 nm that is ascribed to the (002) plane of GaN. Nevertheless, the XRD patterns did not exhibit the usual peaks associated with any of the MCA NPs due to their low concentration of each metal (Table S1, Supporting Information) as determined by inductively coupled plasma‐atomic emission spectroscopy (ICP‐AES). High‐angle annular dark‐field scanning transmission electron microscopy (HAADF‐STEM) characterizations along with corresponding elemental mapping were conducted to investigate the elemental distributions of MCA on GaN NWs (Figure 1d). The resulting images revealed a uniform distribution of iron (Fe), nickel (Ni), chromium (Cr), manganese (Mn), and cobalt (Co) across the sample. Further, the Raman spectra of GaN NWs and MCA/GaN NWs show distinct differences, with the GaN spectrum exhibiting sharp and well‐defined peaks, characteristic of its crystalline structure (Figure S6, Supporting Information). The E₂ (high) mode at ≈570 cm^−^¹ and the A₁(LO) mode at ≈735 cm^−^¹ are prominent in the bare GaN spectrum. In contrast, the multicomponent alloy‐decorated GaN spectrum displays broader peaks with additional features between 800 and 1000 cm^−^¹, indicating lattice disorder and the possible presence of metal oxides due to alloying. These results further suggest the successful decoration of MCA onto GaN.

The photoluminescence (PL) spectra were utilized to study the properties of the catalysts regarding the separation, transport, and recombination processes of electrons and holes. As shown in Figure 1e, the MCA/GaN NWs display a significant reduction in PL intensity compared to bare GaN NWs, suggesting inhibited recombination.^[^ ^18^ ^]^ Moreover, as studied by time‐dependent photoluminescence (TD‐PL) spectroscopy, the recombination rate of photo‐induced charge carriers decreases significantly as the temperature rises on the MCA/GaN (Figure 1f). This suggests that the concentrated illumination‐induced thermal effect enhances the separation of electron–hole pairs, allowing more charge carriers to participate in the reaction. The electron/hole recombination behavior can be further confirmed using Time‐resolution PL (TR‐PL) spectroscopy (Figure 1g). The charge lifetime (τ) of GaN increases from 0.80 to 0.97 ns with the addition of MCA. The findings above indicate that the decoration of MCA onto the surface of GaN NWs is advantageous in the charges behavior for facilitating the subsequent reactions. In addition, the 1D structure of GaN NWs is particularly advantageous for efficient photon absorption by reducing light reflection (Figure S7, Supporting Information) as previously reported.^[^ ^16^, ^19^ ^]^ Overall, the MCA/GaN NWs hybrid exhibit exceptional optical, electrical, and catalytic characteristics making them promise for effective photo‐thermal‐coupled CO_2_ methanation.

**Figure 2:**
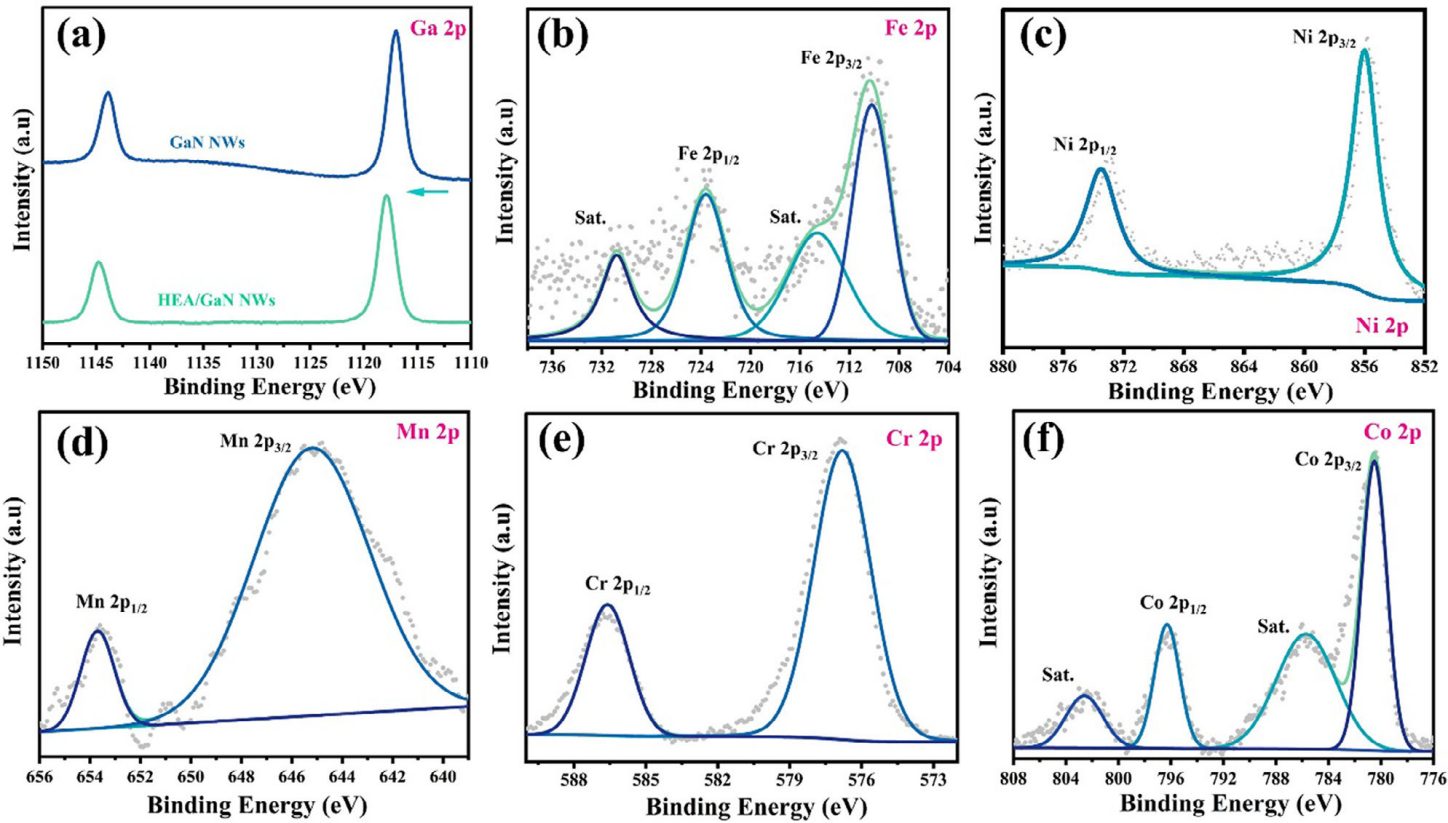
High‐resolution XPS spectra of a) Ga 2P, b) Fe 2p, c) Ni 2P, d) Mn 2P, e) Cr 2P, and f) Co 2P.

To investigate the surface composition and electronic interaction, X‐ray photoelectron spectroscopy (XPS) measurements were carried out (Figure S8, Supporting Information). Typically, the characteristic peaks of Ga 3d and N 1s were located at ≈20 and ≈397 eV, respectively (**Figure** **2**a; Figure S9, Supporting Information).^[^ ^12^ ^]^ Interestingly, the high‐resolution XPS spectra shows a notable shift in the binding energy of Ga 2p and N 1s after introducing MCA, suggesting a redistribution of electron density from GaN to the MCA. For the deposited MCA, it is found that the Fe 2p spectra exhibit multiple peaks characteristic of different oxidation states, suggesting the presence of both Fe^2^⁺ and Fe^3^⁺ (Figure 2b). Similarly, the Ni 2p region displays well‐defined peaks indicative of varied oxidation states (Figure 2c). The Mn 2p spectrum (Figure 2d) presents a broad peak, potentially signifying a mixed oxidation state. The Cr 2p (Figure 2e) and Co 2p regions (Figure 2f) further illustrate sharp peaks corresponding to well‐defined oxidation states. Collectively, these XPS findings elucidate the intricate relationships between the electronic properties of these elements and their collective impact on the catalytic performance in CO₂ hydrogenation, underscoring the importance of structural and electronic modifications imparted by multicomponent alloys in optimizing methane production.

### 2.2 Photo‐Thermal Performance of CO_2_ Hydrogenation to CH_4_

**Figure 3:**
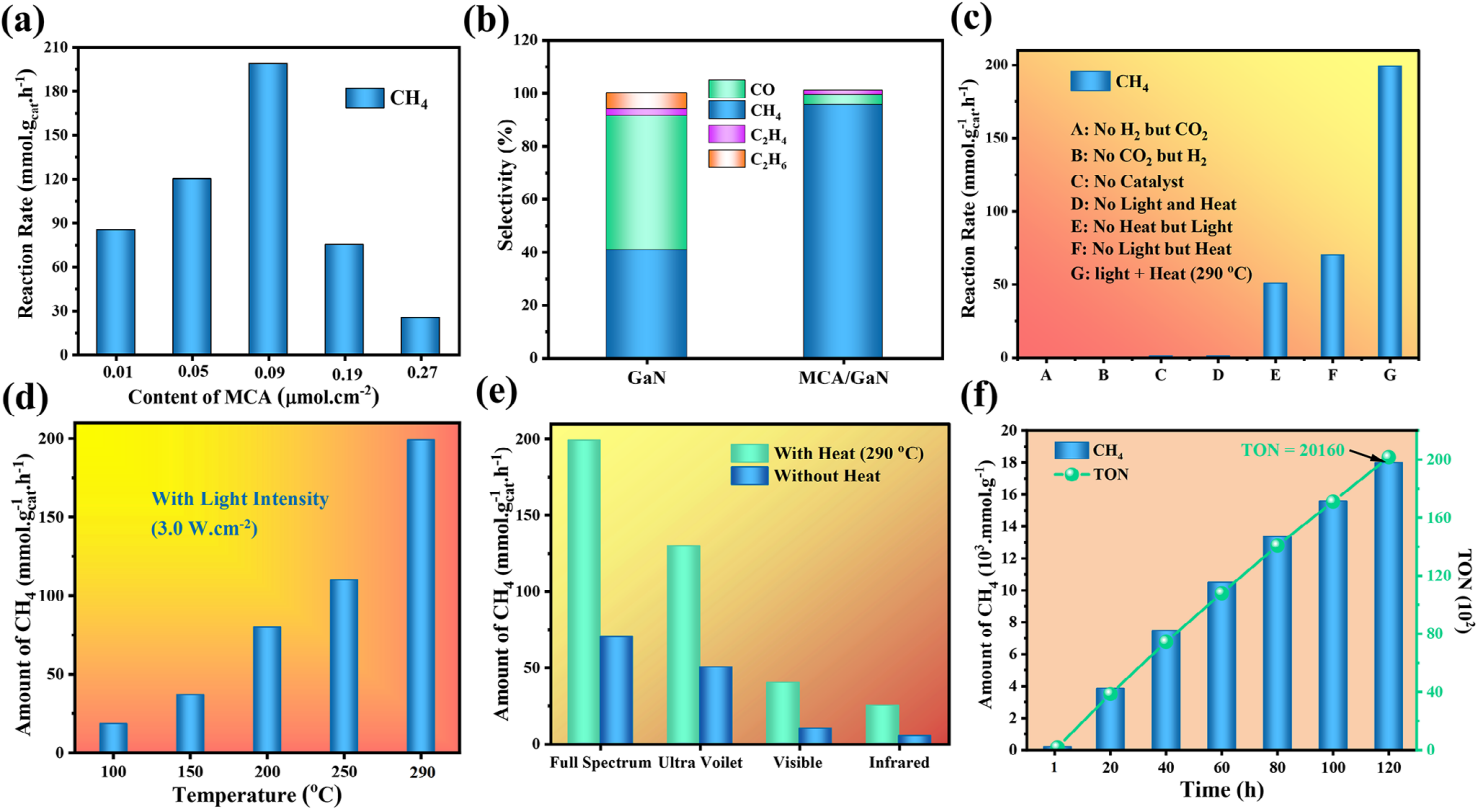
Photo‐thermal‐coupled catalytic activity of CO_2_ methanation over MCA/GaN NWs: a) Influence of the content of MCA on CH_4_ evolution rate from CO_2_ hydrogenation. b) Selectivity of various products over GaN and MCA/GaN NWs, respectively. c) Control Experiment. d) CH_4_ evolution rate at different temperatures under fixed light illumination of 3 W·cm^−2^. e) CH_4_ evolution rate over MCA/GaN NWs under light illumination in different spectral ranges (full spectra, ultraviolet, visible, and infrared) with/without an external heating source; f) Stability test of CO_2_ methanation over MCA/GaN NWs. Experimental Conditions: CO_2_:H_2_ (1:20), 300 W xenon lamp, 3 W·cm^−2^. External Heat 290 °C (The data have a 5–10% error bar during sampling).

The reactions were conducted in a stainless‐steel photo‐thermal‐coupled reactor equipped with a top quartz window using a 300 W xenon lamp as the light source and an external heater. The critical role of MCA was first investigated. As illustrated in **Figure** **3**a, the deposited amount of MCA on the GaN nanowires significantly influenced the catalytic performance, with activity increasing at higher MCA loading concentrations, peaking at 199 mmol·g_cat_^−^¹·h^−^¹ at an optimal loading of 0.09 µmol·cm^−^^2^. When GaN is used alone, CH₄ is produced in limited quantities with lower selectivity (Figure 3b). After loading MCA nanoparticles onto GaN, a substantial improvement in CH₄ selectivity is observed with a marked reduction in CO and other by‐products. This dual improvement underscores the key role of MCA in both amplifying catalytic efficiency and refining product selectivity, making the MCA/GaN composite an effective and selective catalyst for targeted CO₂ methanation.^[^ ^20^ ^]^ Figure 3c provides valuable insights into the performance of CH₄ production under a series of controlled experimental conditions. It is found that CH_4_ indeed originates from CO_2_ hydrogenation using MCA/GaN as the catalyst. More critically, a combined photo‐thermal approach substantially increased the CH_4_ activity compared to photocatalysis and thermos‐catalysis alone. When the incident light intensity is kept unvaried at 3.0 W cm^−^^2^, the CH_4_ activity increases with the increasing reaction temperature (Figure 3d), owing to the enhanced reaction kinetic at higher temperatures. To further elucidate the photo‐thermal‐synergistic effect, the study explored the impact of external heating on CH₄ evolution during CO₂ hydrogenation under various light spectra. As illustrated in Figure 3e, the CH₄ evolution was consistently higher across all light conditions when external heat was applied compared to experiments conducted without heating. This aligns with previous studies, where the photo‐induced thermal effect was shown to play a crucial role in boosting catalytic activity by facilitating more efficient electron–hole separation and surface reaction kinetics.^[^ ^16^, ^19^ ^]^ Interestingly, the application of external heat under UV light alone led to a CH₄ production rate approaching that of the full‐spectrum condition, underscoring the importance role of photoexcited charge carriers in promoting CO_2_ methanation. Without heat, the system's performance under visible and NIR light was much less effective. These findings above suggest the synergetic combination of light spectrum and external heating can significantly improve CH_4_ yield through CO₂ hydrogenation. The stability of this architecture was also evaluated. As shown in Figure 3f, with CH₄ being produced at an average rate of 195 mmol·g^−^¹·h^−^¹, a remarkable turnover number (TON) of 20160 moles of CH₄ per mole of MCA was achieved over 120 h of continuous reaction. Additionally, the variation in turnover frequency (TOF) throughout the 120‐h stability test is not notable (Figure S10, Supporting Information). Following the stability test, the architecture maintained its structural integrity without any significant changes in morphology (Figure S11, Supporting Information) or chemical composition (Figure S12, Supporting Information). However, XPS analysis revealed that MCA deposited on the GaN nanowires diminished after 120 h of operation, which may explain the observed decrease in activity.

**Figure 4:**
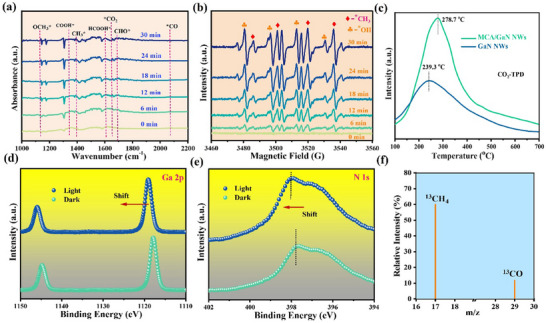
Mechanism Investigation of MCA/GaN NWs for Photo‐thermal‐coupled Catalytic Hydrogenation of CO_2_ toward CH_4_: a) Operando DRIFT spectra of CH_4_ production using MCA/GaN NWs; b) In situ EPR spectra of CH_4_ production over MCA/GaN NWs under a light intensity of 3 W·cm^−2^; c) CO_2_ temperature‐programmed desorption (CO_2_‐TPD) spectra of GaN NWs and MCA/GaN NWs; In situ irradiated XPS of d) Ga 2p and (e) N1s of MCA/GaN NWs; Isotope labeling experiments over MCA/GaN NWs f) Gas Chromatography‐Mass Spectroscopy characterizations of CH_4_ and CO produced from ^13^CO_2_, Experimental conditions: full‐arc 300 W Xenon lamp, light intensity 3 W∙cm^−2^; ^13^CO_2_ atmosphere; H_2_; surface area ≈0.1–0.2 cm^2^; external heat 290 °C.

The source of the superior activity was investigated by conducting correlative characterizations. The in situ diffuse reflectance infrared Fourier‐transform (DRIFT) spectroscopy was first used to examine CO_2_ hydrogenation at the molecular level to reveal the reaction mechanism.^[^ ^21^ ^]^ As shown in **Figure** **4**a, a range of surface‐bound intermediates such as OCH₃*, COOH*, CH₃*, HCOOH*, CHO*, and CO* are progressively detected throughout the reaction. These species suggest a sequential reaction pathway, where CO₂ is initially adsorbed onto the catalyst surface and undergoes hydrogenation, leading to the formation of different intermediates, ultimately resulting in CH₄ production. Notably, the increasing intensity of the CH₃* peak at later time points reflects the formation of methane precursors. The photothermal catalyst, which utilizes both heat and light, significantly enhances the reaction kinetics, thereby accelerating the conversion of CO₂ to CH₄. In addition, the in situ electron paramagnetic resonance (EPR) spectra presented in Figure 4b reveal the dynamic formation of key intermediates, specifically methyl radicals (CH₃*) and hydroxyl groups (OH*), during the CO₂ methanation process under photothermal conditions.^[^ ^22^ ^]^ Over the 30 min, the consistent increase in the intensity of the CH₃* and OH* signals reflects the progressive buildup of these intermediates, confirming their crucial role in the methane formation pathway. The prominent presence of CH₃* suggests that it is a pivotal intermediate in the hydrogenation process, while the observed OH* indicates the involvement of surface hydroxyls, likely facilitating hydrogen transfer. Further, a temperature‐programmed desorption (TPD) characterization was conducted to study the effect of CO_2_ adsorption over the MCA/GaN NWs surface. It shows that MCA/GaN NWs exhibit a significantly stronger CO₂ adsorption capacity compared to bare GaN NWs, as indicated by the higher desorption temperature and larger absorption volume of CO_2_ over MCA/GaN NWs.

The electronic properties of the architecture had a significant impact on the reaction.^[^ ^23^ ^]^ To investigate this effect, in situ irradiated XPS measurements were conducted to monitor the redistribution of photogenerated electrons at the interface between MCA and GaN NWs under light illumination. A slight positive shift in the binding energy of Ga 3d and N 1s was observed (**Figure** **4**d,e). In contrast, a noticeable negative shift in the binding energy of the MCA components (Fe 2p, Co 2p, Mn 2p, Cr 2p, and Ni 2p) was detected (Figure S13, Supporting Information). This shift indicates that, under light illumination, photoexcited electrons are easily transferred from GaN to MCA, facilitated by the strong electronic interaction between the two materials. It is thus favorable for the deep hydrogenation of CO_2_ into CH_4_ owing to the accumulated electrons. An investigation into the origin of carbon‐related products was undertaken through the execution of a ^13^CO_2_ isotope labeling experiment. It is found that the typical feature ^13^CH_4_ was detected by the Gas Chromatography‐Mass Spectroscopy measurement (Shimadzu QP‐2010 GCMS), providing strong evidence that CH_4_ is purely from CO_2_ hydrogenation (Figure 4f). As discussed, the as‐prepared MCA shows a promising cost‐effective alternative cocatalyst for promoting CO_2_ methanation by photo‐thermal‐coupled catalysis.

### 2.3 Theoretical Investigations of CO_2_ Methanation over MCA

**Figure 5:**
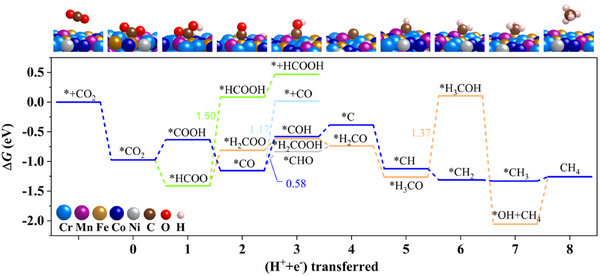
Free energy diagram of CO_2_ reduction on the MCA. The preferred reaction pathway is highlighted with atomistic illustrations of the key intermediates shown at the top.

Density functional theory calculations were conducted to study the reaction mechanism of CO_2_ methanation over MCA at the molecule level. As illustrated in **Figure** **5**, CO_2_ adsorption on the MCA surface is energetically favorable, with a negative Δ*G* of −0.97 eV. In the subsequent step, the formation of *HCOO occurs spontaneously, while the formation of *COOH requires an energy of 0.34 eV. From *HCOO, the generation of HCOOH and CH_4_ demands a Δ*G* of 1.50 and 1.37 eV, respectively, at the potential‐limiting step (PLS). These energies are significantly higher than those required for CH_4_ formation from *COOH, with a Δ*G*_PLS_ of 0.58 eV. Thus, the reaction is more likely to proceed through the pathway via the key intermediate of *COOH. By contrast, the formation of CO gas requires a Δ*G*_PLS_ of 1.17 eV, indicating a preference for CH_4_ over CO, which aligns with the higher yield of CH_4_ observed in the experimental results. From a thermodynamic perspective, the energy barrier for CH₄ formation through the hydrogenation of the HCOO intermediate is 0.58 eV, which is considerably lower than the competing pathways leading to CO (1.17 eV) and HCOOH (1.50 eV). This explains the high selectivity toward methane production observed in the system, as CO and HCOOH formation are kinetically disfavoured.

## 3 Conclusion

In conclusion, integrating MCA composed of Cr, Co, Fe, Ni, and Mn with GaN NWs offers an innovative approach for highly efficient and selective photo‐thermal‐coupled CO₂ hydrogenation toward CH₄. The unique multielement structure of the MCA enabled optimizing the adsorption and activation of CO₂ and H_2_ without noble metals. This hybrid system achieved an impressive methane production rate of 199 mmol·g^−^¹·h^−^¹ with 93.6% selectivity, driven by the synergy between light irradiation and external heating, significantly surpassing the performance of conventional thermo‐catalysis alone. What is more, control experiments, in‐situ spectroscopic characterizations, combined with DFT calculations, revealed that the MCA/GaN system promotes the formation of CH₄. The energy barrier for methane formation was sharply reduced from competing pathways, confirming the thermodynamically and kinetically favorable methane production over CO or HCOOH. This study highlights the potential of integrating MCA as a promising non‐noble cocatalyst with metal nitride for efficient and selective hydrogenation of CO₂ into high‐value fuels and chemicals.

## Conflict of Interest

The authors declare no conflict of interest.

## Author Contributions

M.S.N. and Y.Z. contributed equally to this work. M.S.N. and B.Z. designed the research. M.S.N. conducted the characterizations and performance test. H.Y., W.D., P.W., and X.W. conducted the epitaxial growth. Y.Z. and J.S. contributed to the DFT calculations. J.L. and Z.H. joined the discussion. M.S.N., Y.Z., and B.Z. wrote the paper with the inputs from all other co‐authors. B.Z. led the research.

## Supporting information



Supporting Information

## Data Availability

The data that support the findings of this study are available from the corresponding author upon reasonable request.
