# Supplementary material for: Unlocking Methane Generation via Photo‐Thermal‐Coupled CO2 Hydrogenation by Integrating FeNiCrMnCo Multicomponent Alloy with GaN Nanowires
Source: Adv Sci (Weinh). 2025 Apr 17;12(25):2501298. doi: 10.1002/advs.202501298 (PMC12224925; doi:10.1002/advs.202501298)
Supplement: Supplementary file 1 — Supporting Information [file ADVS-12-2501298-s001.pdf]

ADVANCED SCIENCE
Open Access

Supporting Information

for *Adv. Sci.*, DOI 10.1002/advs.202501298

Unlocking Methane Generation via Photo-Thermal-Coupled CO_2 Hydrogenation by Integrating FeNiCrMnCo Multicomponent Alloy with GaN Nanowires

*Muhammad Salman Nasir, Ying Zhao, Haotian Ye, Jinglin Li, Ping Wang, Ding Wang, Xinqiang Wang, Jun Song, Zhen Huang and Baowen Zhou**

Unlocking Methane Generation via Photo-Thermal-Coupled CO_2 Hydrogenation by Integrating FeNiCrMnCo Multi-Component Alloy with GaN Nanowires

Muhammad Salman Nasir[1+], Ying Zhao[2+], Haotian Ye[3], Jinglin Li[1], Ping Wang[3], Ding Wang[1], Xinqiang Wang[3,5], Jun Song[2], Zhen Huang[1], Baowen Zhou[1]*

1. Key Laboratory for Power Machinery and Engineering of Ministry of Education, Research Center for Renewable Synthetic Fuel, School of Mechanical Engineering, Shanghai Jiao Tong University, 800 Dongchuan Road, Shanghai 200240, China.
2. Department of Mining and Materials Engineering, McGill University, 3610 University Street, Montreal, QC H3A0C9, Canada.
3. State Key Laboratory of Artificial Microstructure and Mesoscopic Physics, School of Physics, Nano-Optoelectronics Frontier Center of Ministry of Education (NFC-MOE), Peking University, Beijing 10087, China.
4. Peking University Yangtze Delta Institute of Optoelectronics, Nantong, Jiangsu 226010, China.
5. Collaborative Innovation Center of Quantum Matter, School of Physics, Peking University, Beijing 100871, China.

* Corresponding authors Email: zhoubw@sjtu.edu.cn;

Methods

MBE growth of GaN NWs.

Catalyst-free gallium nitride nanowires were synthesized on 4-inch silicon (111) wafer using SVT-associated plasma-assisted molecular beam epitaxy (PA-MBE) system under nitrogen-rich conditions. Prior to the growth of GaN nanowires, the Si(111) substrate was outgassed at 800°C for 30 minute, which is helpful for removing any residual oxide layers and contaminants from the substrate surface, ensuring a clean surface for the subsequent growth. Subsequently, the GaN NWs were grown at 600°C. The nitrogen plasma was operated with a radio frequency power of 400 W and a nitrogen flow rate of 1 standard cubic centimeter per minute (sccm) throughout the growth. While the Ga flux was maintained at $5x10^{-7}$ Torr, ensuring a nitrogen-rich growth environment, which is essential for the high-quality growth of GaN nanowires.

Loading of High Entropy Alloys over GaN:

The deposition of high-entropy metals (Fe, Ni, Cr, Co, Mn) was performed in a 0.44 L sealed Pyrex chamber with a quartz lid. The GaN NWs/Si substrate was fixed at the bottom of the chamber. A 60 mL methanol-water solution (CH_3OH/H_2O volume ratio = 1:5) was prepared, and the required amounts of metal precursors ($Fe(NO_3)_3 \cdot 9H_2O$, $Ni(NO_3)_2 \cdot 6H_2O$, $Cr(NO_3)_3 \cdot 9H_2O$, $Co(NO_3)_2 \cdot 6H_2O$, and $Mn(NO_3)_2 \cdot 4H_2O$) all purchased from Shanghai Bailing chemical company, 99.99%) were added sequentially using aqueous solution (0.2 mol/L). Each precursor was added individually, with a 10-minute between additions, ensuring uniform distribution of the metals within the same solution. The total loading amounts and metal ratios were controlled by adjusting the volume of each precursor solution, and the overall metal content was quantitatively measured using ICP-AES characterization. Once all the metals were introduced, the chamber was evacuated, filled with argon to create an inert environment, and irradiated with a 300 W Xe lamp for 30 minutes. After the reaction, the wafer was thoroughly rinsed with distilled water and dried to complete the deposition process.

Characterization

The crystal structure of the samples was analyzed using X-ray diffraction (XRD) patterns obtained from a Bruker D8 Advance diffractometer. The XRD patterns were generated using Cu Kα radiation at 60 kV and 80 mA. The loaded quantity of Ru NPs was determined by measuring the induced coupled plasma (ICP) characterization using an AGILENT ICP-OES 730. The X-ray photoelectron spectroscopy (XPS) analysis was conducted utilizing ESCALAB 250xi equipment with non-monochromatic Al anodes. The C 1s peak at 284.8 eV was employed as the internal standard for the purpose of adjusting the binding energy. The Quattro ESEM (Thermo Fisher) was used to obtain scanning electron microscopy (SEM) pictures. TEM images were obtained using a JEOL 2100F microscope. The Thermo Fisher Scientific Talos F200X S/TEM, operating at 200 kV, was used to acquire high-angle annular dark field-scanning transmission electron microscopy (HAADF-STEM) pictures. The images were obtained using a Super-X EDS detector. The PL and TR-PL spectroscopies were conducted using a FLS980 instrument manufactured by Edinburgh Instruments. The Autosorb-iQ-C

chemisorption analyzer (Quantachrome, USA) was used to evaluate Temperature Programmed Desorption (TPD). The isotope tests were conducted using a TRACE 1310 gas chromatograph that was equipped with a 253Plus mass spectrometry module. The analysis of X-ray photoelectron spectroscopy (XPS) was performed on samples that were irradiated in their original position. The ESCALAB 250xi instrument was used, equipped with non-monochromatic aluminum anodes, and the irradiation was carried out using a 300 W xenon lamp. The Bruker A300 (Germany) was utilized to perform electron paramagnetic resonance (EPR) spectroscopy, specifically in situ electron spin electron spectroscopy. DMPO was employed as a spin trap throughout the analysis. Diffuse reflection Fourier transform infrared (DRIFT) analysis was performed using a Frontier FT-IR Spectrometer, manufactured by PerkinElmer. The spectrometer was fitted with an MCT detector and a 10-cm Demountable Gas Cell. UV-Vis diffuse reflectance spectroscopy (DRS) was recorded on a Shimadzu UV–vis spectrophotometer (UV–2550) with $BaSO_4$ as the background.

Performance evaluation.

Photothermal CO_2 hydrogenation reactions were conducted in a 250 mL photothermal reactor (CE-Au Light, Beijing) with the catalyst placed at the bottom of a metal container inside the reactor (Figure S14). Due to the easily adjustable characteristics of reactant concentration, temperature, and pressure, the evaluation of all catalyst performances was carried out in a conveniently operated atmospheric pressure sealed batch reactor. The introduction of reactants and product analysis were sampled by using syringes. The catalysts were thoroughly washed with deionized water before being fixed at the bottom of the pyrex chamber, followed by vacuum. The reactor was sealed having a small area covered with glass, allowing the 300 W xenon lamp (CE-Au Light, Beijing) to illuminate the system from above (Figure S14). External heating was initiated, and the temperature was raised to 290°C before the lamp was switched on. The chamber was completely evacuated to remove any residual oxygen, ensuring a clean reaction environment. After evacuation, the chamber was filled with high-purity CO_2 (99.9%) and H_2 to a total pressure of 1 atm. Once the system reached the target temperature of 290°C, the xenon lamp was activated, delivering 3 W/cm^2 light intensity to the catalyst through the glass lid. The reaction progress was monitored, and readings were recorded at specific time intervals to track the hydrogenation of CO_2 under these photothermal conditions. The gaseous products underwent analysis using a gas chromatograph (GC-9080, Sun) that was outfitted with a thermal conductivity detector (TCD) and a flame ionization detector (FID).

The product evolution rate, TOF, and TON were calculated according to the following equations:

$$\text{Gas evolution rate}=\frac{\text{Gas yield per unit area of catalyst per hour}}{\text{Mass of catalyst per unit area}} \quad (1)$$

$$\text{Mass of catalyst per unit area} = L \times v \times \rho \quad \mathbf{(2)}$$

Where the L is determined as the length of GaN NWs, the v is denoted as the fill factor based on the top-view SEM image of GaN NWs on Si substrate, and ρ is the density of GaN NWs that is 6.1 $g \cdot cm^{-3}$.

$$\text{TOF} = \frac{\text{Gas evolution rate (μmole)}}{\text{Content of immobilized MCA NPs (μmole) x Reaction Time (h)}} \quad \mathbf{(3)}$$

$$\text{TON} = \frac{\text{Gas production amount (μmole)}}{\text{Content of immobilized MCA NPs}} \quad \mathbf{(4)}$$

Computational methods.

Many studies[1] have investigated the crystal structure of the muti metals alloy, consistently indicating that it adopts a face-centered cubic (FCC) structure. Therefore, we initially constructed a 3 × 3 × 5 FCC bulk supercell with nine atoms of each element randomly distributed, using the lattice parameter of nickel, as Ni is stable in the FCC phase. After relaxation, the bulk structure yielded a lattice constant of 3.50Å, which closely matches the previously reported value of 3.59 Å[1d]. The relaxed bulk was subsequently cleaved into a three-layer slab for adsorption calculations.

All calculations were performed using spin-polarized density functional theory (DFT) calculations within the Vienna Ab Initio Simulation Package (VASP)[2]. The projector-augmented wave (PAW) method was adopted to describe the interaction between valence electrons and ions[3]. Perdew-Burke-Ernzerhof (PBE) functional was employed to present the exchange-correlation in the Kohn-Sham equation[4]. Van der Waals (vdW) interactions were included using Grimme's DFT-D2 method[5]. A plane-wave basic set with a kinetic energy cutoff of 400 eV was used. For all slabs, the Brillion zone was sampled using 4 × 4 × 1 Gamma-centered grid of k-points. All structures converged until the atomic forces and the total energies reached 0.05 eV/Å and 10^{-4} eV, respectively.

The free energies were computed using the computational hydrogen electrode (CHE) model[6]. The Gibbs free energy of adsorption ΔG was calculated by:

$$\Delta G = E_{ad} + \Delta ZPE - T\Delta S \quad \mathbf{(5)}$$

where E_{ads} is the adsorption energy defined by:

$$E_{ads} = E_{surface+adsorbate} - E_{surface} - E_{adsorbate} \quad \mathbf{(6)}$$

ΔZPE and ΔS represent the changes in zero-point energy and entropy, respectively. The temperature, denoted as T, was set to the room temperature.

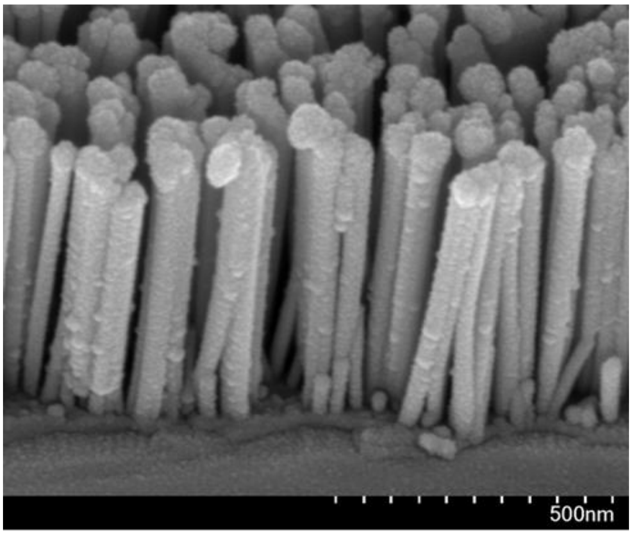

Figure S1: (a) A 25° tilted-view SEM image of MCA/GaN NWs

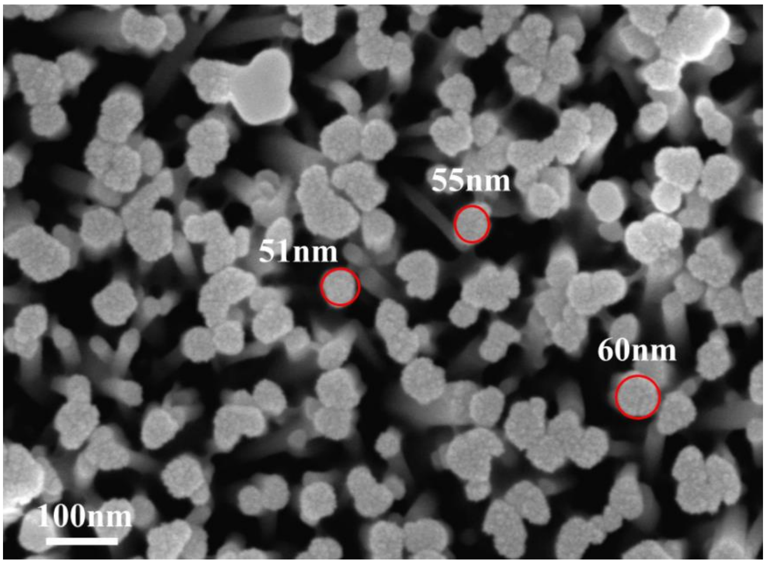

Figure S2: A top-view SEM image of MCA/GaN NWs; red circle shows the diameter of NWs

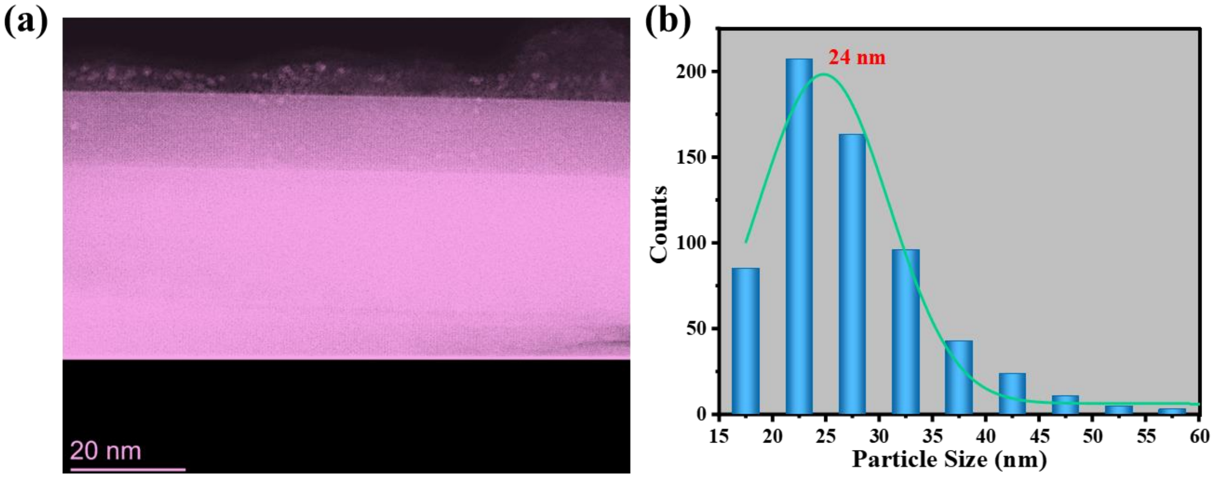

Figure S3: (a) A TEM image of MCA/GaN NWs; (b) The size contribution of MCA nanoparticles.

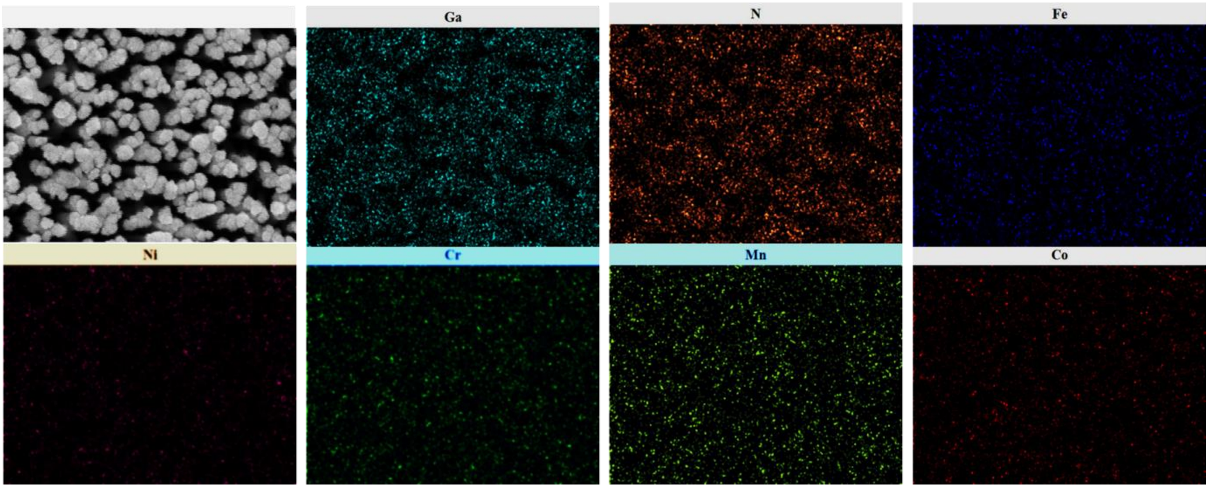

Figure S4: Energy Dispersive X-ray Spectroscopy (EDX) images of FeNiCrMnCo-decorated GaN NWs.

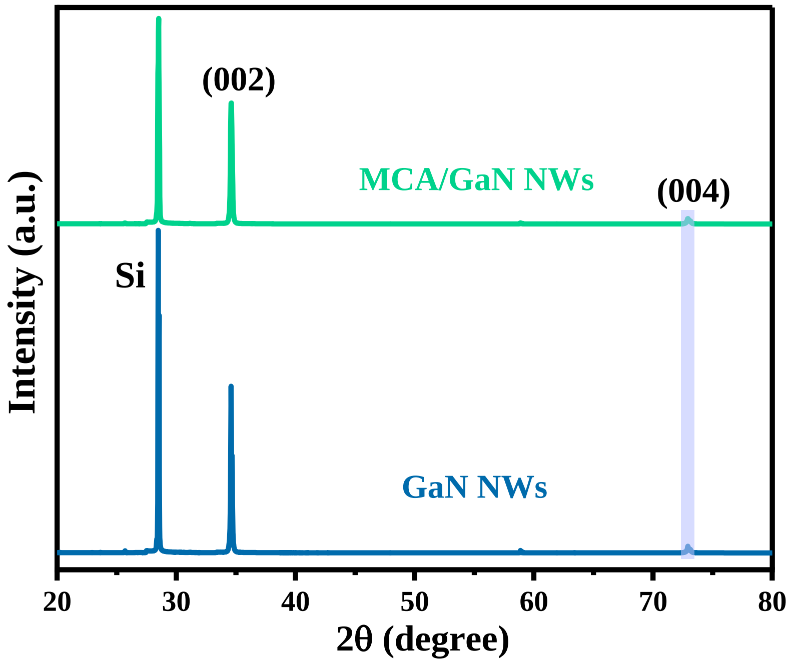

Figure S5 : XRD patterns of GaN NWs, and MCA/GaN NWs

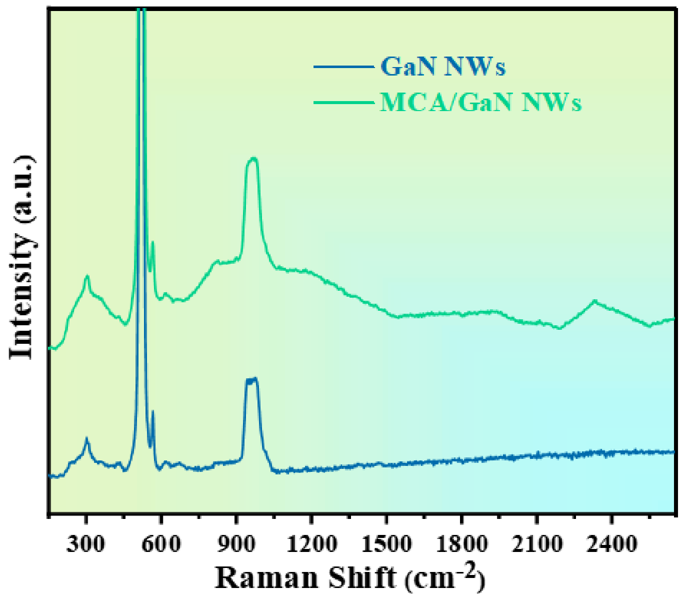

Figure S6: Raman Spectroscopy of GaN NWs, and MCA/GaN NWs

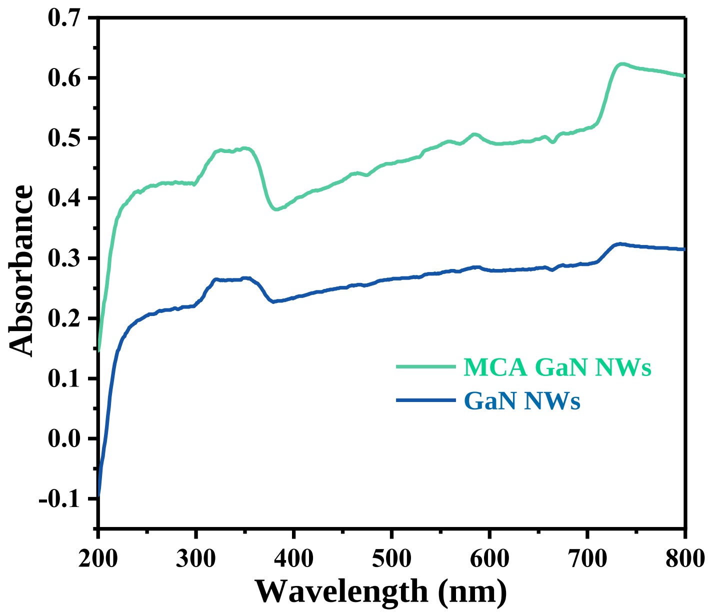

Figure S7: Ultraviolet-visible reflectance spectroscopy analysis of GaN NWs and MCA/GaN NWs.

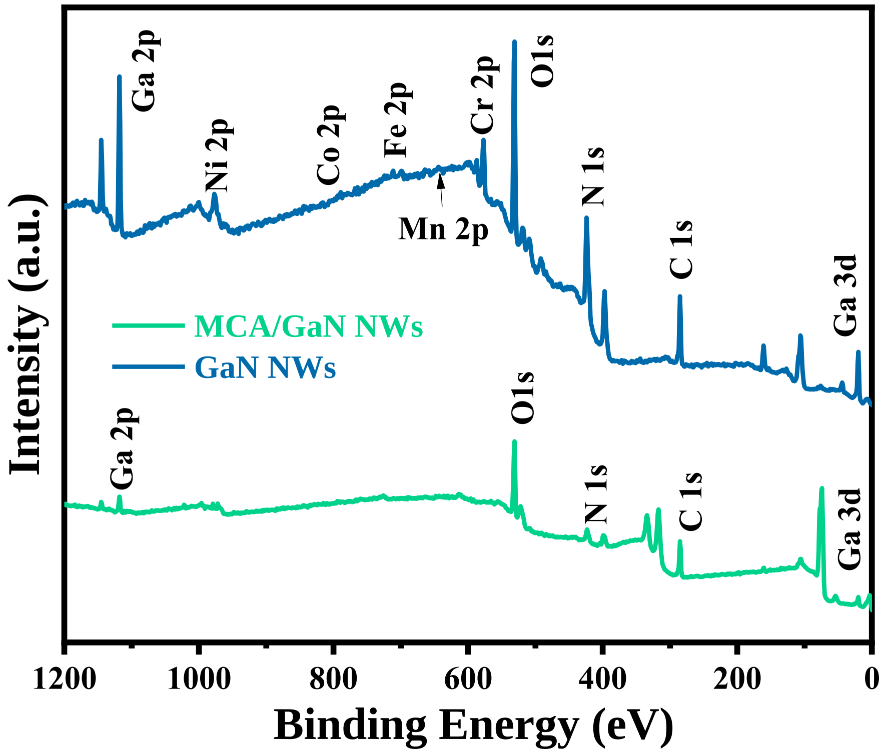

Figure S8: XPS survey of GaN NWs and MCA/GaN NWs

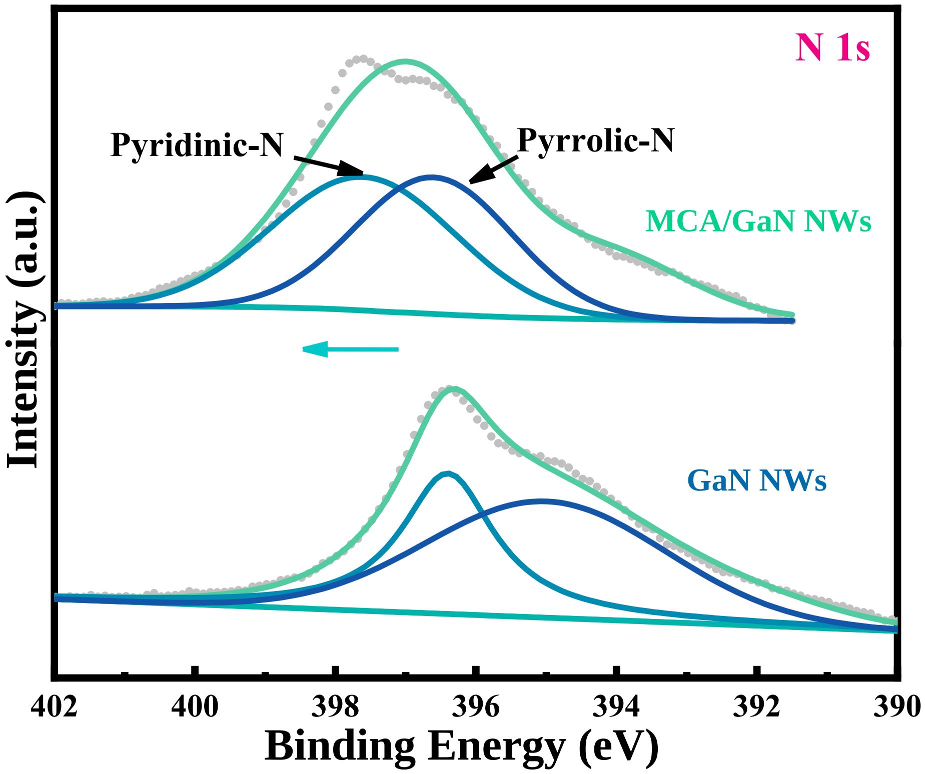

Figure S9: HR-XPS of N 1s spectra

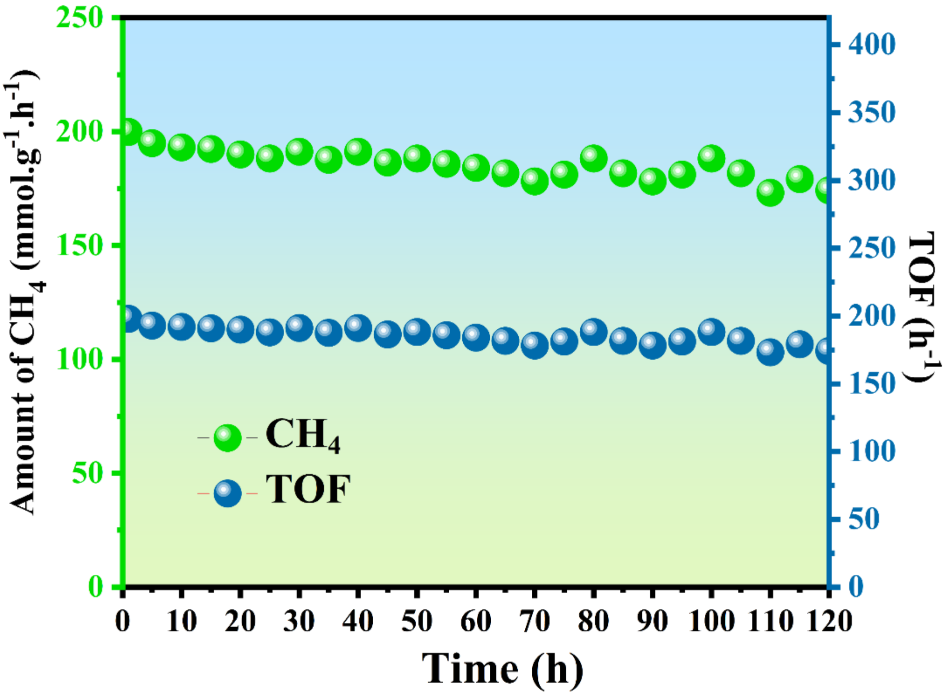

Figure S10: CH_4 evolution rate and TOF during the stability test up to 120 hrs. Experimental conditions: full-arc 300 W Xenon lamp, light intensity 3 W·cm^{-2}; atmospheric CO_2:H_2; wafer surface area ~0.1-0.2 cm^2; External Heat: 290 ºC.

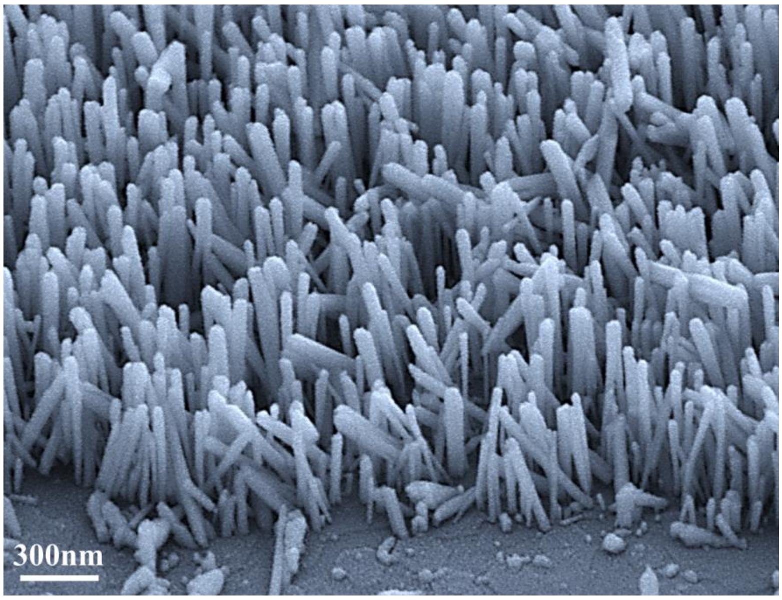

Figure S11: SEM image of MCA supported by GaN NWs after stability test.

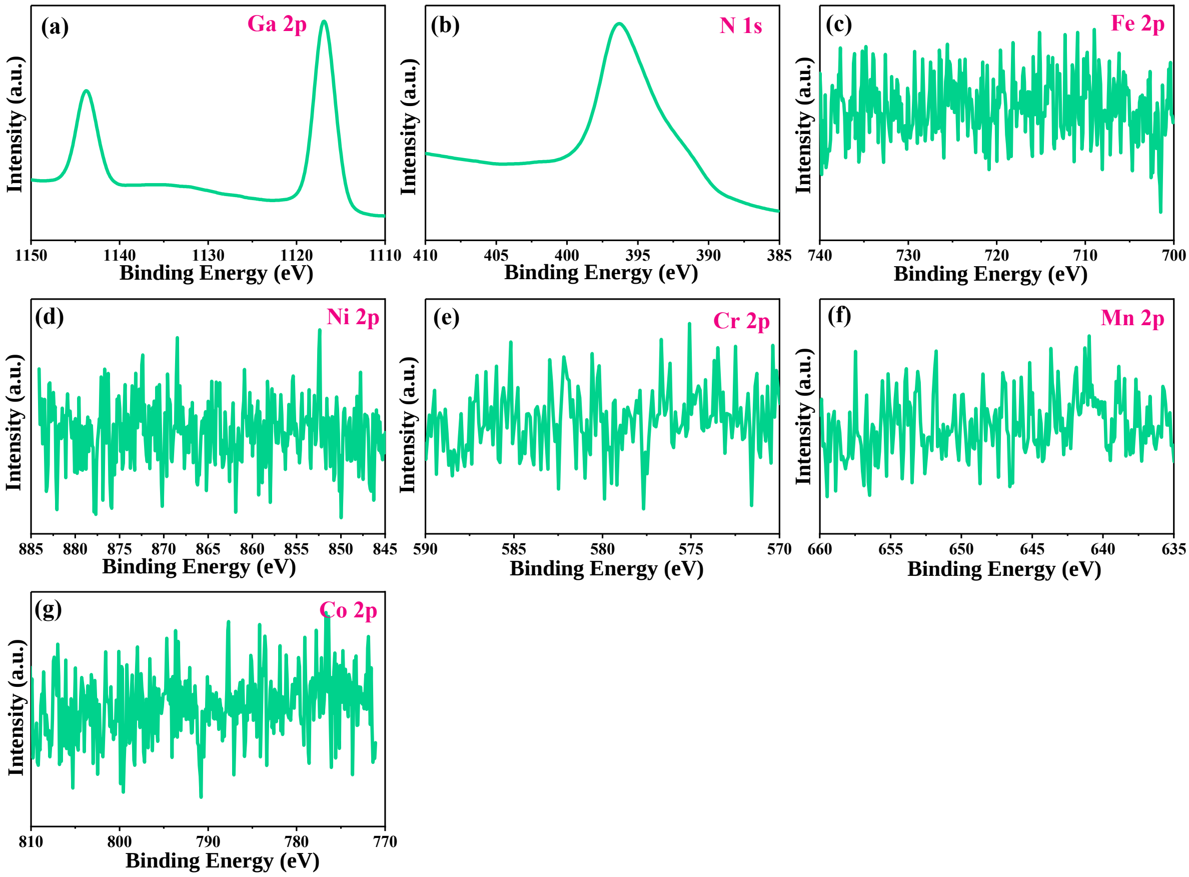

Figure S12: HR-XPS of MCA/GaN NWs individual metal after stability test (a) Ga 2p (b) N1s; (c) Fe 2p; (d) Ni 2p (e) Cr 2p; (f) Mn 2p (g) Co 2p

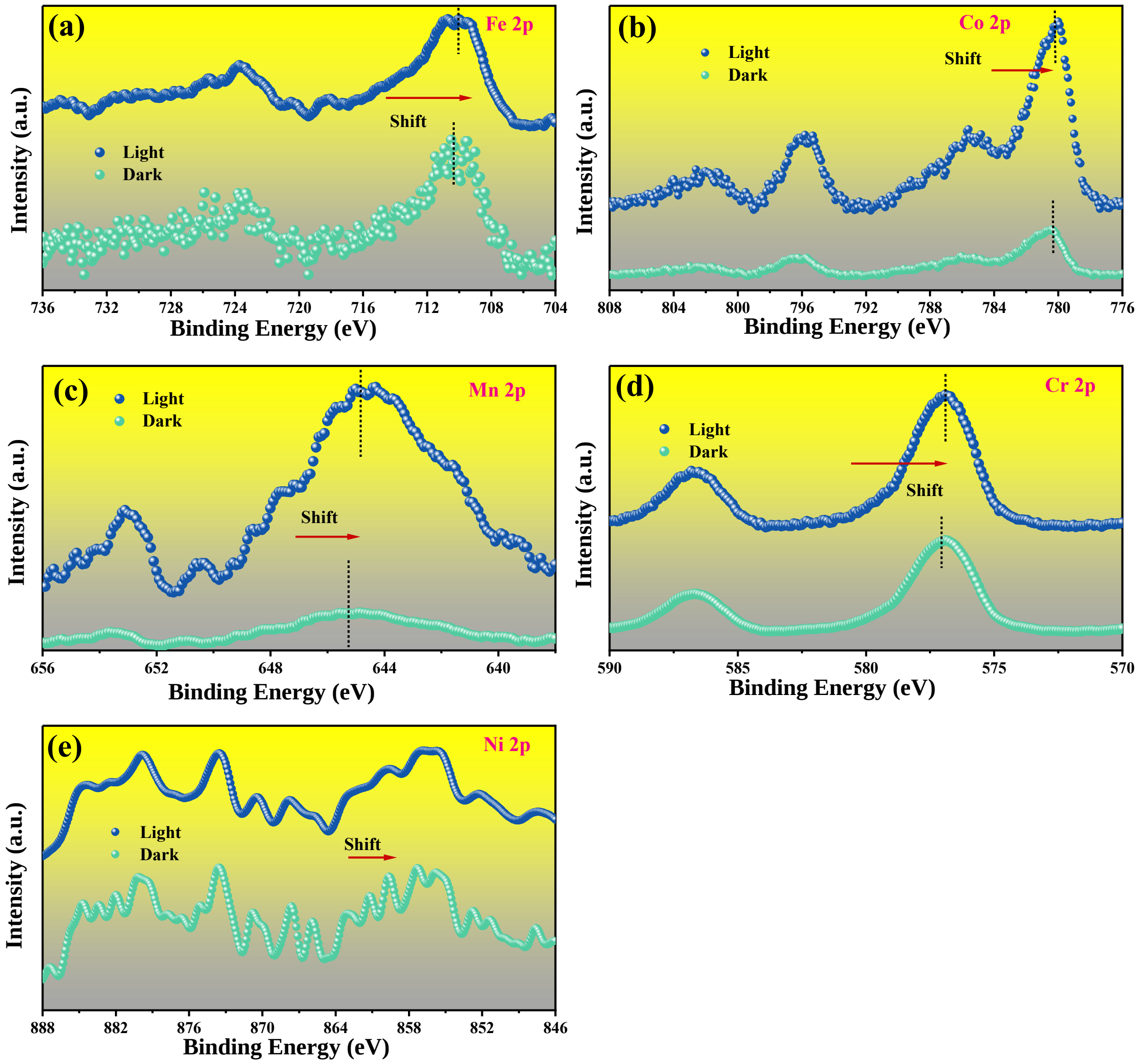

Figure S13: *In situ* irradiated XPS of (a) Fe 2p (b) Co 2p; (c) Mn 2p; (d) Cr 2p (e) Ni 2p;

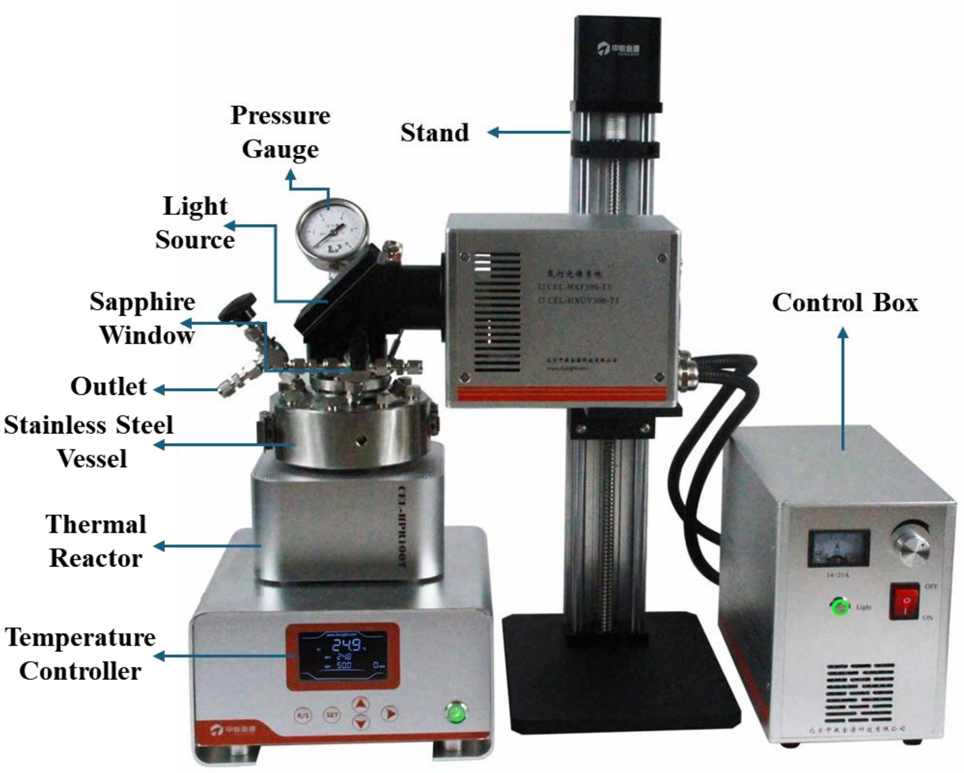

Figure S14: Digital photo of the Photo-Thermal Reactor setup (Credit: CE-Au Light,Beijing)

Table S1 The measured amounts of (FeNiCrMnCo) in the optimized MCA/GaN NWs.

Amount ($\mu mol \cdot cm^{-2}$)					Total ($\mu mol \cdot cm^{-2}$)
Fe	**Ni**	**Cr**	**Mn**	**Co**	
0.0388	**0.0143**	**0.0107**	**0.0155**	**0.0132**	**0.093**

Table S2 The comparison of the related catalyst for the CH_4 production using Photothermal method with this work.

Catalyst	Experimental Conditions	CH_4 Activity (mmol/g/h)	CH_4 Selectivity	Stability	Reference
FeNiCrMnCo/GaN	0.1 MPa, 290 ºC, H_2/CO_2 = 10, light	199	93	120	This work
Au/Ce0 .95 Ru 0.05O_2	72% H_2, 18% CO_2, 10% Ar, light	473	100	12	[7]
Ru@FL LDH	0.1 MPa, 350 ºC, H_2/CO_2 = 4, light	277	100	12	[8]
Ru/Al_2 O_3	0.1 MPa, 350 ºC, 15.5% CO_2, 80.9% H_2, light	0.146	100	15	[9]
Ni/CeO_2	0.1 MPa, 225 ºC, H_2/CO_2 =4 light	147	97	150	[10]
NiO@ Si_XNS	0.1 MPa, 300 ºC, H_2/CO_2 =4, light	0.10	91	12	[11]
Ru@N $i_2V_2O_7$	0.1 MPa, 350 ºC, H_2/CO_2 = 4 , light	115	99	20	[12]

References:

[1] aK. M. Bertsch, K. E. Nygren, S. Wang, H. Bei, A. Nagao, *Corrosion Science* **2021**, *184*, 109407; bC. Chu, W. Chen, Z. Chen, Z. Jiang, H. Wang, Z. Fu, *Acta Metallurgica Sinica (English Letters)* **2021**, *34*, 445-454; cB. T. Tran, H. A. Pham, V. T. Nguyen, D. P. Doan, T. T. T. Hoang, N. X. Ho, D. V. Nguyen, T. Le Manh, *Journal of Alloys and Compounds* **2024**, *973*, 172860; dL. Patriarca, A. Ojha, H. Sehitoglu, Y. I. Chumlyakov, *Scripta Materialia* **2016**, *112*, 54-57.

[2] aG. Kresse, D. Joubert, *Physical Review B* **1999**, *59*, 1758-1775; bG. Kresse, J. Furthmüller, *Physical Review B* **1996**, *54*, 11169-11186.

[3] P. E. Blöchl, *Physical Review B* **1994**, *50*, 17953-17979.

[4] aJ. P. Perdew, K. Burke, M. Ernzerhof, *Physical Review Letters* **1996**, *77*, 3865-3868; bJ. P. Perdew, J. A. Chevary, S. H. Vosko, K. A. Jackson, M. R. Pederson, D. J. Singh, C. Fiolhais, *Physical Review B* **1992**, *46*, 6671-6687.

[5] S. Grimme, *Journal of Computational Chemistry* **2006**, *27*, 1787-1799.

[6] J. K. Nørskov, J. Rossmeisl, A. Logadottir, L. Lindqvist, J. R. Kitchin, T. Bligaard, H. Jónsson, *The Journal of Physical Chemistry B* **2004**, *108*, 17886-17892.

[7] H. Jiang, L. Wang, H. Kaneko, R. Gu, G. Su, L. Li, J. Zhang, H. Song, F. Zhu, A. Yamaguchi, *Nature Catalysis* **2023**, *6*, 519-530.

[8] J. Ren, S. Ouyang, H. Xu, X. Meng, T. Wang, D. Wang, J. Ye, *Advanced Energy Materials* **2017**, *7*.

[9] S. Chen, A. M. Abdel-Mageed, M. Dyballa, M. Parlinska-Wojtan, J. Bansmann, S. Pollastri, L. Olivi, G. Aquilanti, R. J. Behm, *Angewandte Chemie International Edition* **2020**, *59*, 22763-22770.

[10] Y. Xie, J. Chen, X. Wu, J. Wen, R. Zhao, Z. Li, G. Tian, Q. Zhang, P. Ning, J. Hao, *ACS Catalysis* **2022**, *12*, 10587-10602.

[11] X. Yan, W. Sun, L. Fan, P. N. Duchesne, W. Wang, C. Kübel, D. Wang, S. G. H. Kumar, Y. F. Li, A. Tavasoli, *Nature Communications* **2019**, *10*, 2608.

[12] Y. Chen, Y. Zhang, G. Fan, L. Song, G. Jia, H. Huang, S. Ouyang, J. Ye, Z. Li, Z. Zou, *Joule* **2021**, *5*, 3235-3251.
